# Brain circadian clock neurons drive fitness advantages in *Drosophila*

**DOI:** 10.1016/j.isci.2026.117125

**Published:** 2026-08-10

**Authors:** Sae Aikawa, Shoichiro Tamura, Makiko Mimura, Taishi Yoshii

**Affiliations:** 1 Graduate School of Environmental, Life, Natural Science and Technology, Okayama University, Okayama 700-8530, Japan

**Keywords:** activity rhythms, clock neurons, *Drosophila*, adaptive advantage, reproductive success, resonance hypothesis

## Abstract

The adaptive significance of circadian clocks is widely assumed due to their ubiquity; yet, direct empirical evidence remains scarce. Evaluating these benefits is often confounded by pleiotropic effects in conventional circadian null mutants. To address this, we selectively altered the circadian period exclusively within brain clock neurons in *Drosophila melanogaster*. Multi-generational competition assays revealed that flies with aberrant rhythms exhibit a significant fitness disadvantage under standard light-dark (LD 12:12) cycles. This disadvantage was abolished under constant light, confirming that the selection pressure is specifically mediated by the circadian clock. Furthermore, paternity assays conducted under LD 12:12 indicated that the timing of brain clock neurons influences male reproductive success, providing a potential mechanistic link between clock-controlled behavior and fitness. Intriguingly, we found that these fitness costs are highly photoperiod-dependent. Under short-day conditions (LD 8:16), the short-period strain (*dbt*^*S*^) maintained a significantly higher overall frequency than the long-period strain (*dbt*^*L*^). Our behavioral observations suggest that this difference may be associated with the quality of activity rhythms; specifically, *dbt*^*S*^ lacked defined morning peaks and showed suppressed nocturnal activity, potentially narrowing its window for reproductive interactions compared to *dbt*^*L*^. These findings illustrate that the circadian system does not merely track a 24-h cycle but functions as a flexible feature that enables flies to cope with changing day lengths by aligning their mating behavior with the most favorable time of day.

## Highlights

• Brain-specific clock manipulation reveals adaptive value of circadian rhythms
• Wild-type rhythms confer a competitive advantage in standard light-dark cycles
• Selective disadvantage is abolished in constant light, confirming clock effects
• Neuronal clock speed optimizes reproductive success via phase synchronization

## Graphical abstract

**Figure:**
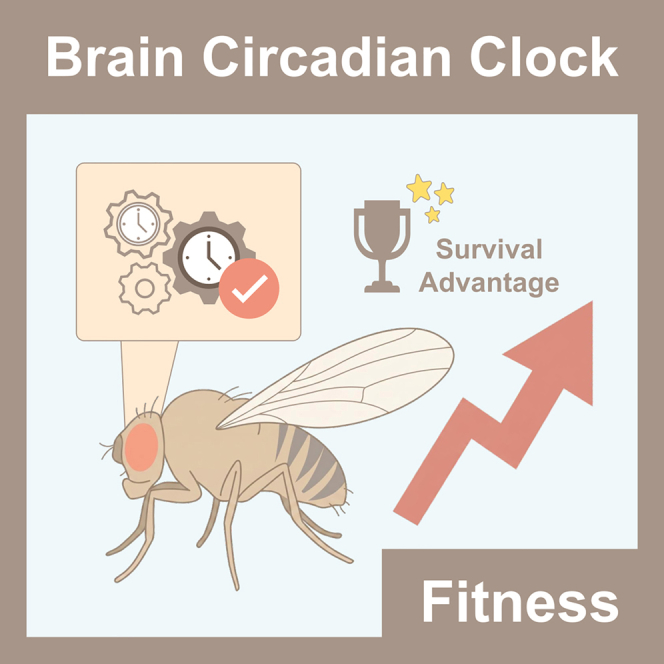

## Teaser

Neurogenetics; Evolutionary biology; Chronobiology

## Introduction

The circadian clock, an endogenous timing system operating on a near-24-h cycle, orchestrates a vast array of physiological and behavioral processes to ensure temporal alignment with the external environment. In humans, the fundamental importance of this system is underscored by the severe health consequences of its disruption, ranging from metabolic syndromes to mood disorders.^1^

In animal models, circadian locomotor activity serves as a robust behavioral proxy for the clock’s functional output. *Drosophila melanogaster* has been a very important model for deciphering these mechanisms, exhibiting bimodal locomotor activity peaks under light-dark (LD) conditions and self-sustained free-running rhythms in constant darkness (DD).^2^^,^^3^^,^^4^ At the core of this system for controlling the behavioral rhythms is a network of approximately 240 clock neurons organized into distinct clusters.^5^^,^^6^^,^^7^ The approximately 240 clock neurons are organized into nine distinct clusters, including the small and large ventrolateral neurons (s-LN_v_ and l-LN_v_), the 5^th^ LN_v_, dorsal lateral neurons (LN_d_), lateral posterior neurons, and dorsal neurons 1, 2, and 3. While individual clock neuron clusters, particularly dorsal neuron 1, contribute to daily sleep/wake cycles, the s-LN_v_s, 5^th^ LN_v_, and LN_d_s are the most critical for generating the two characteristic activity peaks.^8^^,^^9^^,^^10^^,^^11^^,^^12^

Although the molecular mechanisms of the circadian clock are well understood, how the clock contributes to evolutionary fitness under complex ecological conditions remains unclear. Population genetic studies and laboratory selection experiments in *Drosophila* have clearly demonstrated that the evolution of the circadian clock is driven by environmental adaptation.^13^^,^^14^ Clock disruption is known to negatively impact various fitness-related traits, including adult lifespan,^15^^,^^16^^,^^17^ pre-adult developmental duration,^18^ lifetime fecundity,^17^ larval growth rate,^19^ and sperm count.^20^ Using competition assays, Horn et al.^21^ demonstrated the fitness advantages of a functional circadian clock; wild-type flies outcompeted various clock mutants, such as period null (*per*^*01*^), short-period (*per*^*S*^), and long-period (*per*^*L*^) mutants, under LD 12:12 conditions. Similar findings have been reported in cyanobacteria, *Arabidopsis thaliana*, and rodents,^22^^,^^23^^,^^24^^,^^25^ suggesting that the absence of proper circadian rhythms confers a competitive disadvantage. Specifically, Horn et al. showed that the fertility and offspring survival rates of *per* mutants were significantly lower than those of wild-type flies. However, since the *per* gene is known to have pleiotropic effects unrelated to the circadian clock,^20^^,^^26^^,^^27^^,^^28^^,^^29^^,^^30^ caution is warranted when concluding that the circadian clock itself inherently confers a survival advantage.

Notwithstanding these extensive studies, the functional importance of circadian timing remains unclear. The primary obstacle is pleiotropy: mutations that alter clock timing often disrupt other physiological processes, making it difficult to isolate the clock’s specific contribution to fitness. Separating the adaptive benefits of timing from pleiotropic artifacts is crucial yet remains an unresolved hurdle. Here, we overcome this limitation by utilizing a tissue-specific approach to isolate the neural mechanisms of circadian fitness. The *double-time* (*dbt*) gene encodes a vital kinase that regulates the stability of the PERIOD protein through phosphorylation, thereby serving as a key determinant of the pace of the core molecular feedback loop. By selectively overexpressing *dbt* alleles in specific subsets of brain clock neurons, we generated transgenic fly lines with altered circadian periods while maintaining functional molecular oscillators in peripheral tissues, thereby minimizing systemic pleiotropic effects. These lines were subjected to long-term competition assays against *white*^*1118*^ (*w*^*1118*^) control flies spanning 27 generations. To directly test the role of the clock, we used constant light (LL) as a control condition to disrupt circadian rhythms without altering the genetic background.^31^ We demonstrate that the observed fitness advantage is specifically tied to circadian rhythmicity rather than non-circadian gene functions. Furthermore, paternity assays were employed to investigate the mechanistic basis of this advantage, revealing that the speed of the neuronal oscillator directly influences reproductive success. Taken together, our results show that the brain’s internal clock is a key trait for survival. We demonstrate that its true value lies in matching the timing of behavior with environmental rhythms. This study provides a clear answer to the long-standing debate over pleiotropy by showing that the speed of brain clock neurons is directly linked to evolutionary fitness.

## Results

### Generation and characterization of *DvPdf-dbt* transgenic fly lines

**Figure 1:**
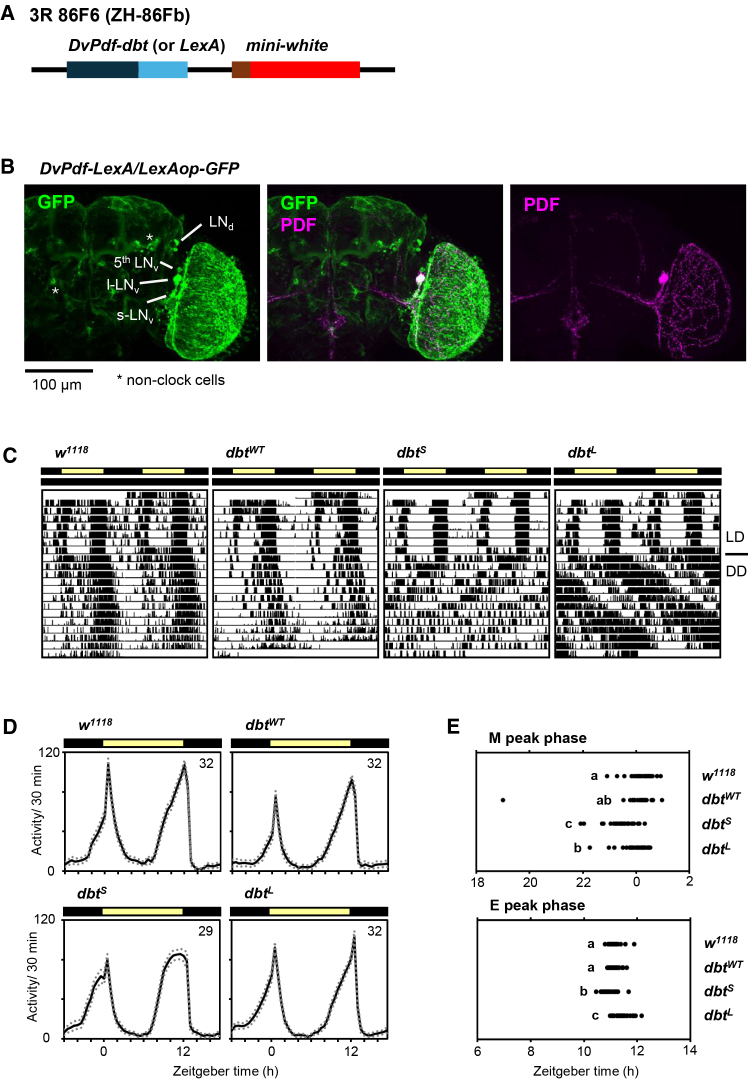
Circadian characteristics of brain-specific *dbt*-expressing flies (A) Schematic of the *DvPdf-dbt* and *mini-white* transgenes. Dark and light blue indicate the upstream and coding regions of *dbt*, respectively, while dark and light red indicate those of *mini-white*. Transgenes were integrated into the 86F6 site on chromosome 3 R using the PhiC31 system. (B) Expression patterns of GFP (green) and PDF (magenta) in the adult brain. Images show the *DvPdf-LexA/LexAop-GFP* line. GFP is expressed in s-LN_v_s, l-LN_v_s, the 5^th^ LN_v_, and LN_d_s, in addition to several cells (asterisks) outside the circadian network. Scale bars, 100 μm. (C) Locomotor activity rhythms under LD 12:12 and constant darkness (DD). Representative actograms are shown for *w*^*1118*^ and *dbt* transgenic lines. Flies were entrained for seven days under LD 12:12 before being transferred to DD. (D) Mean daily activity profiles (± SEM) under LD 12:12. The number of flies (*n*) is indicated in the upper right of each figure. White and black bars above the graphs represent the light and dark phases, respectively. (E) PHASE analysis was used to evaluate the phase of the morning (M) and evening (E) activity peak. The phase of each activity peak is represented by individual dots. Different letters indicate significant differences between strains (*p* < 0.01, Mann-Whitney U test followed by pairwise comparisons with Holm’s adjustment).

To manipulate the speed of the circadian clock specifically within brain neurons, we utilized the upstream regulatory region of the *Drosophila virilis Pigment-dispersing factor* (*DvPdf*) gene in combination with various *dbt* mutant alleles^32^^,^^33^^,^^34^ (Figure 1A). This ∼1.9-kb *DvPdf* promoter fragment contains specific *cis*-regulatory elements that drive expression in the s-LN_v_s and l-LN_v_s, the 5^th^ LN_v_, and a subset of the LN_d_s. Of these, the s-LN_v_s, the 5^th^ LN_v_, and the LN_d_s are recognized as the critical pacemakers governing locomotor activity rhythms. Importantly, the *DvPdf-gal4* driver is reported to lack expression in peripheral tissues,^35^ thereby minimizing systemic pleiotropic effects that could confound fitness assessments. We generated four transgenic lines—*DvPdf-dbt*^*WT*^ (*dbt*^*WT*^; wild-type control), *DvPdf-dbt*^*S*^ (*dbt*^*S*^; short-period), *DvPdf-dbt*^*L*^ (*dbt*^*L*^; long-period), and *DvPdf-LexA*—all integrated into the same genomic locus (3 R, ZH-86Fb) via PhiC31 integrase-mediated transgenesis to eliminate positional effects. To verify the expression specificity, brains from *DvPdf-LexA/LexAop-GFP* flies were immunostained with anti-GFP and anti-PDF antibodies (Figure 1B; Figure S1). Consistent with previous findings,^32^ GFP expression was localized to the s-LN_v_s, l-LN_v_s, 5^th^ LN_v_, and LN_d_s. In addition to the clock neurons, GFP expression was detected in several non-clock cell clusters. One cluster exhibiting robust GFP signal was situated near the mushroom body. Two other clusters, showing weak expression, were identified lateral and ventral to the mushroom body and in the proximity of the lateral horn. Another weak cluster was located along the rim of the optic lobes. Furthermore, strong GFP signals were observed in the abdominal ganglion cells of the ventral nerve cord, specifically the PDF abdominal neuromere (PDFAb) neurons.^36^ To mitigate genetic background effects, each line was outcrossed to *w*^*1118*^ for six generations.

Behavioral analysis under constant darkness (DD) confirmed that *dbt*^*S*^ and *dbt*^*L*^ flies exhibited significantly shortened and lengthened free-running periods compared with that of *dbt*^*WT*^ flies, respectively (Figure 1C). The rhythm strength (power) of *dbt*^*S*^ flies was lower than that of *dbt*^*WT*^, and a small number of *dbt*^*S*^ individuals exhibited complex rhythms (Figure 1C; Figure S2). These observations are partly consistent with the previous report on *DvPdf-GAL4/UAS-dbt*^*S*^ flies by Yao et al. (2016).^37^ However, in contrast to the period variability of *DvPdf-GAL4/UAS-dbt*^*S*^ flies reported in their study, most of our *dbt*^*S*^ flies showed consistent free-running periods. This discrepancy likely reflects different expression levels between the GAL4-UAS system and our direct promoter-based system. Under LD 12:12 conditions, the morning and evening peaks of *dbt*^*S*^ flies were significantly phase-advanced compared to those of *w*^*1118*^ and *dbt*^*WT*^ controls (Figures 1D and 1E), with advances of approximately 30 min and 15 min, respectively. In contrast, *dbt*^*L*^ flies exhibited a significant phase delay (approximately 15 min) only in the evening peak, likely due to the overriding influence of light-on masking effects.

### Normal circadian rhythms enhance survival rates under LD12:12

**Figure 2:**
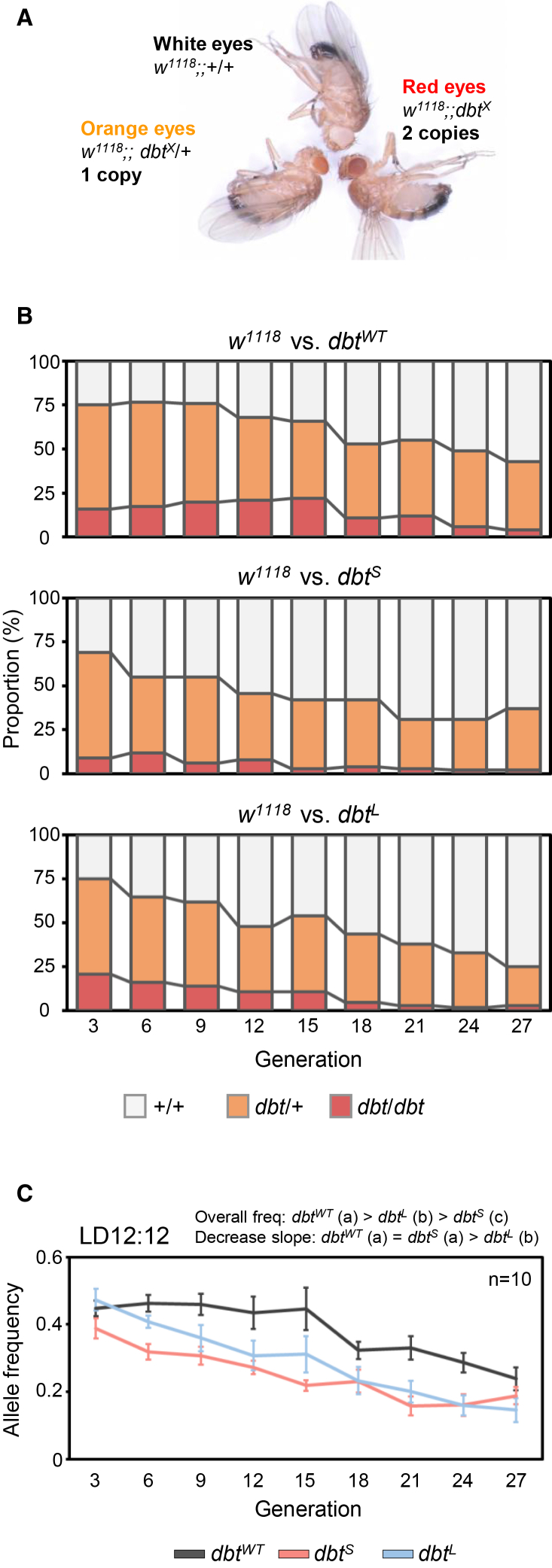
Competition assays under LD 12:12 conditions (A) Eye color phenotypes associated with each genotype. Orange eyes indicate a single copy of the *dbt* transgene (*dbt/* + ; heterozygous), while red eyes indicate two copies (*dbt/dbt*; homozygous). Flies without the transgene exhibit white eyes (+/+). (B) Temporal changes in genotype proportions over 27 generations. The percentages of individuals with each eye color—white (+/+), orange (*dbt/* + ), and red (*dbt/dbt*)—are shown as the pooled population from ten replicate vials. The competition was initiated in generation 1 using heterozygous *dbt/* + males and females. Genotypes were scored every three generations. (C) Mean (± SEM) allele frequency of the *dbt* transgene across 27 generations. Black, light red, and light blue lines represent *dbt*^*WT*^, *dbt*^*S*^, and *dbt*^*L*^, respectively (*n* = 10 vials for each line). Different letters in the upper right of the graph indicate significant differences between strains for overall allele frequencies and decrease slopes (*p* < 0.05, GLMM followed by pairwise contrasts with Holm’s adjustment).

Genotypes were distinguished by eye color: homozygous flies carrying two copies of the *dbt* transgene exhibit red eyes due to the dosage of the *mini-white* marker, *while* heterozygotes carrying a single copy exhibit orange eyes (Figure 2A). In these competition assays, each *dbt* transgenic line was reared in competition with *w*^*1118*^ flies across multiple generations under LD 12:12 conditions (Figure S3). To ensure sufficient time for detecting selection pressure, we tracked the populations for 27 generations (approximately one year), conducting a census every three generations (Figure 2B). This duration was determined based on previous research indicating that 20 generations were sufficient to demonstrate the fitness disadvantages of *per* mutants relative to wild-type flies.^21^ To avoid the effects of the transgenes, we compared the three *dbt* transgenic lines that share the same genetic background except for the *dbt* alleles, instead of comparing them directly to *w*^*1118*^ mutants. Thus, *dbt*^*WT*^ served as an appropriate reference line, allowing us to accurately assess the relative performances of *dbt*^*S*^ and *dbt*^*L*^ based solely on their clock-period phenotypes (Figure 2C). At the midpoint (generation 15), *dbt*^*S*^ and *dbt*^*L*^ exhibited significantly lower allele frequencies than *dbt*^*WT*^ (β = −0.628 ± 0.035 and −0.457 ± 0.034, respectively; both *p* < 0.001). This corresponds to 1.87-fold (*dbt*^*S*^) and 1.58-fold (*dbt*^*L*^) higher odds of carrying wild-type allele than *dbt*^*S*^ or *dbt*^*L*^ at the mean generation. Moreover, *dbt*^*L*^ declined significantly faster than *dbt*^*WT*^ over generations (interaction: β = −0.025 ± 0.004, *p* < 0.001), indicating a progressive fitness loss of *dbt*^*L*^ compared to *dbt*^*WT*^. Across 27 generations, all *dbt* alleles declined in frequency (β = −0.046 ± 0.003, *p* < 0.001), suggesting that the transgene insertion itself imposes a slight disadvantage compared to *w*^*1118*^. Nonetheless, *dbt*^*WT*^ allele frequencies fluctuated around the initial 0.5 during the early phase of the assay (generations 3–15), showing occasional significant deviations (gen 3 and 12), but without a sustained shift as assessed by GLMM-based tests against the expected frequency of 0.5. A clearer decline emerged after generation 18 (0.32 at gen 18 and 0.31 at gen 21; both *p* < 0.001), whereas allele frequencies in *dbt*^*S*^ and *dbt*^*L*^ deviated significantly from 0.5 by generation 6. These results demonstrate that both lengthening and shortening of the circadian period impair reproductive fitness, either through an accelerated exclusion or through a persistently reduced population prevalence.

### Constant light abolishes clock function, neutralizing the impact of period length on survival

**Figure 3:**
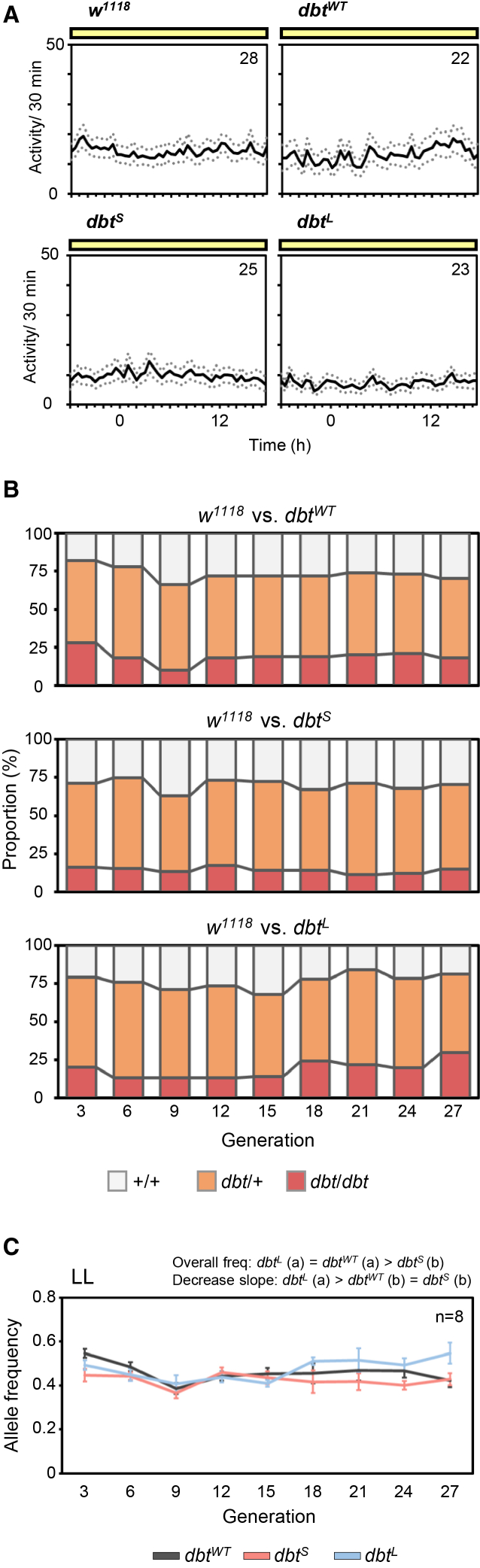
Competition assays under LL conditions (A) Mean daily activity profiles (± SEM) under LL. The number of flies (*n*) is indicated in the upper right of each figure. (B) Temporal changes in genotype proportions under LL over 27 generations. The percentages of individuals with each eye color—white (+/+), orange (*dbt/* + ), and red (*dbt/dbt*)—are shown as the pooled population from eight replicate vials. (C) Mean (± SEM) allele frequencies of the *dbt* transgenes across 27 generations. Black, light red, and light blue lines represent *dbt*^*WT*^, *dbt*^*S*^, and *dbt*^*L*^, respectively (*n* = 8 vials for each line). Different letters in the upper right of the graph indicate significant differences between strains for overall allele frequencies and decrease slopes (*p* < 0.05, GLMM followed by pairwise contrasts with Holm’s adjustment).

The LD12:12 competition assay results suggest that the circadian period influences fitness. However, these survival differences might stem from pleiotropic effects of *dbt* overexpression rather than the circadian period itself. As all *dbt* transgenic lines exhibit arrhythmicity under LL (Figure 3A; Figure S4), we repeated the competition assays in LL. This experimental design allows us to confirm whether the observed fitness decline reflects circadian misalignment or non-circadian pleiotropy. Strikingly, the selective disadvantage observed in LD was effectively abolished or significantly altered under LL (Figures 3B and 3C). The general rate of decline for all transgenic lines was substantially reduced (from β = −0.046 in LD to β = −0.008 in LL). Notably, the fitness disadvantage of *dbt*^*L*^ flies disappeared under LL, with no further evidence of a steeper decline compared to *dbt*^*WT*^. Similarly, while the overall frequency of *dbt*^*S*^ flies remained slightly lower than that of the control, they maintained a stable population trajectory over 27 generations, with a rate of decline indistinguishable from *dbt*^*WT*^ (Figures 3B and 3C). These results show that the fitness costs of *dbt* mutations depend on the function of the circadian clock.

### General fitness and life-history traits remain unaffected across *dbt* transgenic lines

**Figure 4:**
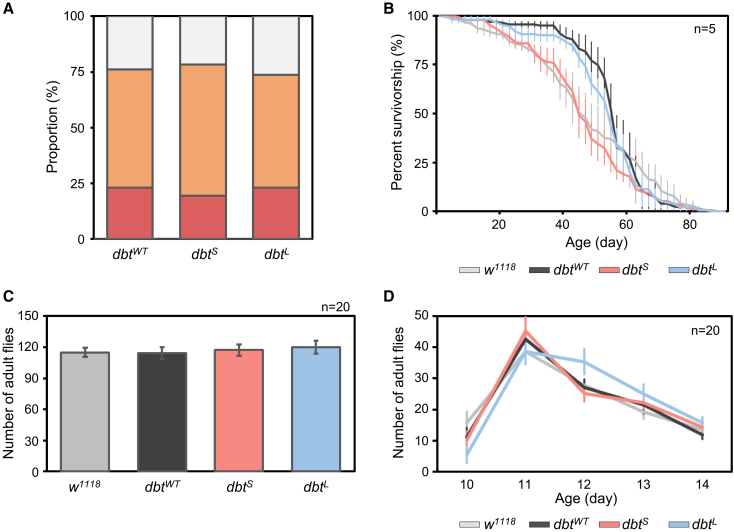
Fundamental physiological and developmental characteristics under LD 12:12 (A) Offspring genotype proportions from *dbt/* + parents. The percentages of each genotype—white ( + */* + ), orange (*dbt/* + ), and red (*dbt/dbt*)—are shown as the pooled population from ten replicate vials. This assay confirms the Mendelian inheritance and viability of each transgene line. (B) Lifespan of *w*^*1118*^ and *dbt* homozygous males. Data represent mean (± SEM) lifespan. For each genotype, five replicate vials were used, each containing 30 males (*n* = 150 per genotype). (C) Reproductive output (Fecundity). The mean (± SEM) number of adult offspring produced by four females of each genotype is shown (*n* = 20 vials per genotype). (D) Developmental eclosion profile. The mean (± SEM) number of flies eclosing daily from day 10–14 after egg-laying is shown (*n* = 20 vials per genotype).

To identify the factors underlying the observed fitness decline, we evaluated several life-history traits of the *dbt* flies. First, we assessed the population frequency in the first generation, which comprised the offspring of *dbt/* + parents. While only males were monitored in the long-term assays to track phenotypic frequency, both males and females were included in this initial assessment. In the first generation, all *dbt* transgenic lines exhibited a frequency of approximately 25% (Figure 4A), consistent with the expected Mendelian ratios. This result confirms a balanced starting population and suggests that the selection pressure against these flies acts gradually, rather than through immediate lethality.

We also measured the lifespan of each *dbt* line (Figure 4B) and found that their longevity was comparable to, or exceeded, that of the *w*^*1118*^ control. In our competition assay protocol, young adults are allowed to mate and oviposit for seven days before being removed from the vials. Since the lifespans of all strains significantly exceeded this seven-day window, differential mortality is unlikely to be the primary driver of selection in the competition assays. Notably, *dbt*^*WT*^ and *dbt*^*L*^ strains exhibited longer lifespans than *w*^*1118*^ and *dbt*^*S*^ strains. This suggests that the rescue of the *white* gene and circadian rhythmicity may interact in a complex manner to influence longevity. Furthermore, we quantified the number of offspring produced between days 10 and 14 post-oviposition. Total offspring counts did not differ between strains (Figure 4C), nor were there any detectable differences in the timing of adult emergence (Figure 4D). Taken together, these results indicate that the *dbt* lines do not suffer from general defects in fecundity or developmental viability.

### Circadian clock speed is a critical determinant of male reproductive fitness

**Figure 5:**
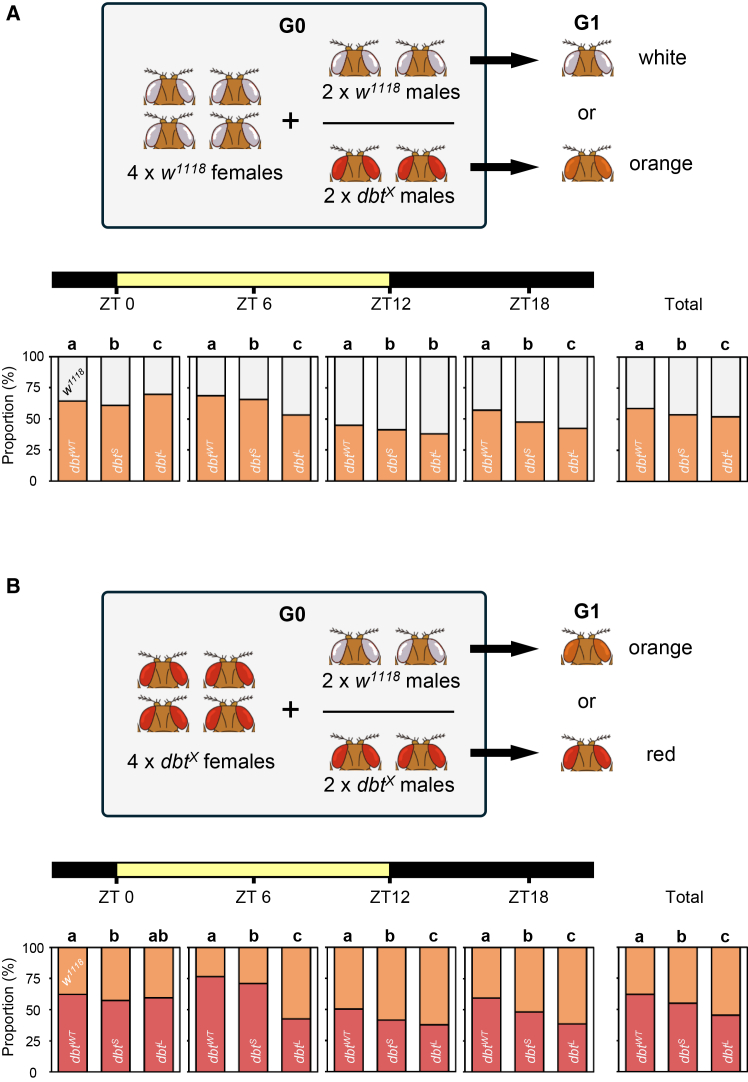
Male reproductive success (paternity) under LD 12:12 conditions (A) Proportion of offspring sired by experimental males in competition with *w*^*1118*^ males. Four *w*^*1118*^ females were paired with two *w*^*1118*^ and two *dbt* homozygous males at four zeitgeber times (ZT0, ZT6, ZT12, and ZT18; *n* = 20 vials per genotype at each time point). The “Total” graph represents data pooled across all four time points. (B) Four *dbt* homozygous females were paired with two *w*^*1118*^ and two *dbt* homozygous males. Different letters indicate significant differences between strains within each ZT (*p* < 0.05, GLMM followed by pairwise comparisons with Holm’s adjustment).

Fitness differences could emerge from multiple stages, ranging from mating preferences to adult developmental viability. To specifically isolate reproductive success while accounting for both mating preference and post-mating competition, we conducted paternity assays under LD 12:12 conditions. Since mating behavior in *Drosophila* is under circadian control,^38^^,^^39^^,^^40^^,^^41^ we initiated these assays at four different zeitgeber times (ZT 0, 6, 12, and 18). The results revealed that the reproductive success of each male genotype fluctuated significantly depending on the time of pairing (Figures 5A and 5B). For example, with *w*^*1118*^ females (Figure 5A), *dbt*^*L*^ males were more successful when the assay began at ZT0 (estimated probability 0.71, *p* < 0.001), but their success rate declined at ZT12 (0.36, *p* = 0.009) and marginally at ZT18 (0.43, *p* = 0.12), consistent with the expected phase shifts in their circadian mating rhythms. This pattern was even more pronounced when *dbt*^*X*^ females were used (0.38, *p* < 0.001 at ZT12 and 0.37, *p* < 0.001 at ZT18) based on a binomial generalized linear mixed model. When the data from all four time points were combined (referred to as Total in Figure 5), the male’s internal clock speed emerged as a highly significant predictor of siring success, which resulted in reduced reproductive success compared to *dbt*^*WT*^ males (*dbt*^*S*^: β = −0.25 with *w*^*1118*^ females and β = −0.33 with *dbt*^*X*^ females; *dbt*^*L*^: β = −0.34 with *w*^*1118*^ females and β = −0.74 with *dbt*^*X*^ females; all *p* < 0.001), while all *dbt*^*X*^ males had statistically higher or equal reproductive success compared to *w*^*1118*^ males when encountering started during day time (i.e., ZT0 and ZT6). Post-hoc pairwise comparisons confirmed a consistent hierarchy of competitive advantage: *dbt*^*WT*^ > *dbt*^*S*^ > *dbt*^*L*^ (*p* < 0.001) when ZT6, ZT12 and ZT18. With *w*^*1118*^ females, *dbt*^*WT*^ males were approximately 1.28 and 1.41 times more likely to sire offspring than *dbt*^*S*^ and *dbt*^*L*^ males, respectively (Figure 5A). This advantage became even more pronounced when paired with *dbt* females, where *dbt*^*WT*^ males sired 1.39 times more offspring than *dbt*^*S*^ males and 2.11 times more than *dbt*^*L*^ males (Figure 5B). Together, these findings demonstrate that the speed of the male neuronal oscillator is a critical and determinant of reproductive fitness.

It is important to consider that eye color may also influence female mating preference. In the assays involving the *dbt*^*WT*^ control, offspring sired by red-eyed *dbt*^*WT*^ males were outnumbered by white-eyed offspring at ZT0 and ZT6, whereas the proportion of offspring from white-eyed *w*^*1118*^ males increased at ZT12 and ZT18. This pattern suggests a light-dependent preference for red-eyed males during the day, while eye color appears to have less influence on selection during the night.

To test whether these time-dependent reproductive advantages require a functional circadian clock, we next performed paternity assays under LL conditions (Figure S5). Since distinct zeitgeber times do not exist in LL, the flies were paired at a single time point (12:00 p.m.). As expected, the distinct competitive hierarchy observed in LD were largely abolished or altered under LL. With *w*^*1118*^ females, the previous clear advantage of *dbt*^*WT*^ males disappeared; no statistically significant differences were detected between *dbt*^*WT*^ and *dbt*^*S*^ males (*p* = 0.0671), nor between *dbt*^*WT*^ and *dbt*^*L*^ males (*p* = 0.4393). Furthermore, when paired with *dbt*^*X*^ females, the differences among the three genotypes were completely neutralized. These results demonstrate that the reproductive advantages observed in LD conditions are driven by the circadian clock. Interestingly, while *w*^*1118*^ females showed almost no preference between white-eyed and red-eyed males under LL, *dbt*^*X*^ females exhibited a clear preference for red-eyed males. This suggests that red-eyed females preferentially select males with the same red-eye phenotype, possibly because differences in eye pigmentation alter visual perception, making red-eyed males more conspicuous or attractive.

### Fitness costs are modulated by the photoperiod

*Drosophila* exhibit a bimodal activity pattern characterized by morning and evening peaks,^4^^,^^42^ which adapt flexibly to varying photoperiods.^43^^,^^44^^,^^45^^,^^46^ Importantly, the intrinsic period of the circadian clock dictates the phase of locomotor activity relative to environmental cycles. When the phase control governed by this intrinsic period significantly misaligns with photoperiods, this temporal mismatch may impose fitness costs. To investigate how these phase differences influence competitive fitness, we evaluated the relative performance of *dbt*^*S*^ and *dbt*^*L*^ by competing each strain against the *w*^*1118*^ control under LD 16:8 and LD 8:16.

**Figure 6:**
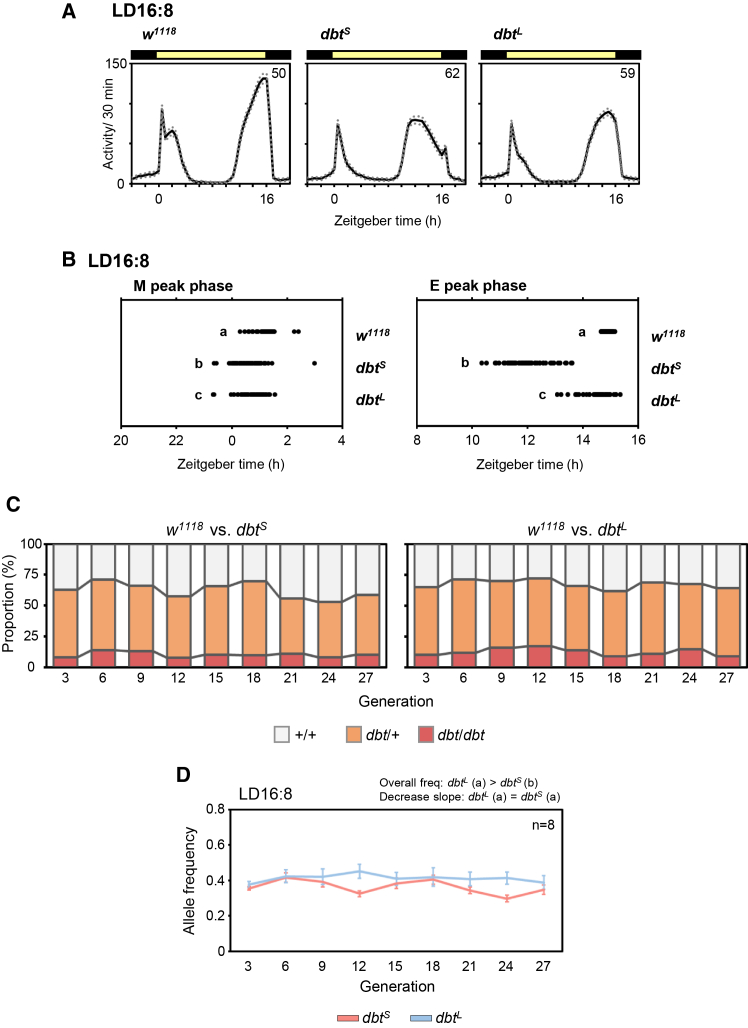
Competition assays under long-day (LD 16:8) conditions (A) Mean daily activity profiles (± SEM) under LD 16:8. The number of flies (*n*) is indicated in the upper right of each figure. (B) PHASE analysis was used to evaluate the phase of the morning (M) and evening (E) activity peak. The phase of each activity peak is represented by individual dots. Different letters indicate significant differences between strains (*p* < 0.01, Mann-Whitney U test followed by pairwise comparisons with Holm’s adjustment). (C) Temporal changes in genotype proportions under LD 16:8 over 27 generations. The percentages of individuals with each eye color—white (+/+), orange (*dbt/* + ), and red (*dbt/dbt*)—are shown as the pooled population from eight replicate vials. (D) Mean (± SEM) allele frequencies of the *dbt* transgenes across 27 generations. Light red and light blue lines represent *dbt*^*S*^ and *dbt*^*L*^, respectively (*n* = 8 vials for each line). Different letters in the upper right of the graph indicate significant differences between strains for overall allele frequencies and decrease slopes (*p* < 0.05, GLMM followed by pairwise contrasts with Holm’s adjustment).

Under long-day conditions (LD 16:8), *w*^*1118*^ control flies exhibited a morning peak following lights-on and an evening peak prior to lights-off (Figure 6A). Although the morning peak phases of *w*^*1118*^, *dbt*^*S*^, and *dbt*^*L*^ flies were significantly different from one another, the peak in *dbt*^*L*^ flies was not delayed relative to the *w*^*1118*^ control (Figure 6B). These phase differences between strains were within a range of approximately 10–20 min. In *dbt*^*S*^ flies, the evening peak was markedly advanced (approximately 3 h) compared to *w*^*1118*^, occurring well before lights-off. In contrast, the evening phase of *dbt*^*L*^ flies showed a smaller advance (approximately 20 min) relative to the control. Consequently, the average difference in the evening peak phase between *dbt*^*S*^ and *dbt*^*L*^ flies was approximately 2.5 h. Competition assays under these LD 16:8 conditions revealed that the allele frequencies of both *dbt*^*S*^ and *dbt*^*L*^ remained relatively stable over 27 generations (Figures 6C and 6D). Notably, *dbt*^*L*^ maintained a slightly but significantly higher overall frequency than *dbt*^*S*^ (at gen 15, β = −0.191 ± 0.036 for *dbt*^*S*^ relative to *dbt*^*L*^, *p* < 0.001; odds ratio *dbt*^*L*^/*dbt*^*S*^ = 1.21). This indicates that under long-day conditions, *dbt*^*L*^ possessed a slight competitive advantage over *dbt*^*S*^.

**Figure 7:**
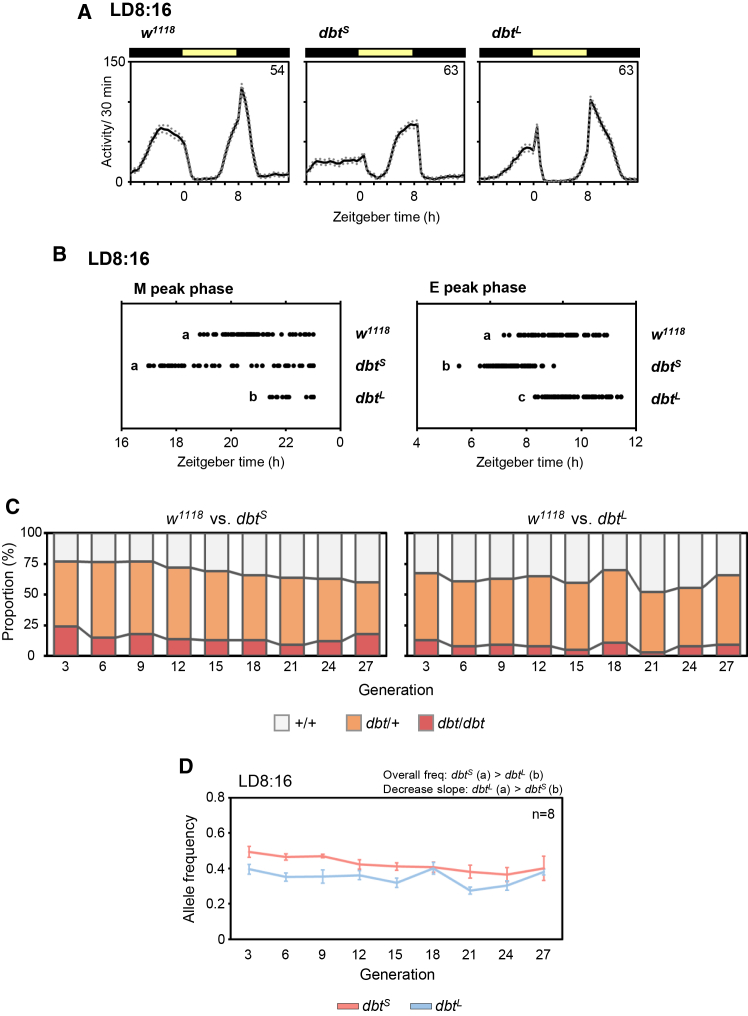
Competition assays under short-day (LD 8:16) conditions (A) Mean daily activity profiles (± SEM) under LD 8:16. The number of flies (*n*) is indicated in the upper right of each figure. (B) PHASE analysis was used to evaluate the phase of the morning (M) and evening (E) activity peak. The phase of each activity peak is represented by individual dots. Different letters indicate significant differences between strains (*p* < 0.01, Mann-Whitney U test followed by pairwise comparisons with Holm’s adjustment). (C) Temporal changes in genotype proportions under LD 8:16 over 27 generations. The percentages of individuals with each eye color—white (+/+), orange (*dbt/* + ), and red (*dbt/dbt*)—are shown as the pooled population from eight replicate vials. (D) Mean (± SEM) allele frequencies of the *dbt* transgenes across 27 generations. Light red and light blue lines represent *dbt*^*S*^ and *dbt*^*L*^, respectively (*n* = 8 vials for each line). Different letters in the upper right of the graph indicate significant differences between strains for overall allele frequencies and decrease slopes (*p* < 0.05, GLMM followed by pairwise contrasts with Holm’s adjustment).

Under short-day conditions (LD 8:16), *w*^*1118*^ control flies exhibited a morning peak occurring well in advance of lights-on and an evening peak coinciding with the lights-off transition (Figures 7A and 7B). In *dbt*^*S*^ flies, the morning peak was diminished or absent, whereas the evening peak was significantly advanced (approximately 80 min) compared to *w*^*1118*^. Conversely, *dbt*^*L*^ flies showed significant phase delays in both morning and evening peaks relative to *w*^*1118*^ (approximately 2 h and 45 min, respectively). Consequently, the average difference in the evening peak phase between *dbt*^*S*^ and *dbt*^*L*^ flies was approximately 2 h. Competition assays under LD 8:16 revealed that although the allele frequencies of both *dbt*^*S*^ and *dbt*^*L*^ declined significantly (both *p* < 0.001), their survival dynamics were distinct (Figures 7C and 7D). While *dbt*^*S*^ maintained a higher overall frequency throughout the experiment (at gen 15, β = −0.364 ± 0.035 for *dbt*^*L*^ relative to *dbt*^*S*^, *p* < 0.001; odds ratio *dbt*^*S*^*/dbt*^*L*^ = 1.44), *dbt*^*L*^ exhibited a significantly slower rate of decline compared to *dbt*^*S*^ (genotype × generation interaction: z = 3.28, *p* = 0.001). Taken together, these findings demonstrate that the fitness costs associated with altered circadian periods are significantly modulated by the environmental photoperiod.

## Discussion

Circadian clocks are nearly ubiquitous across diverse taxa, suggesting that they have evolved to provide significant adaptive advantages.^47^^,^^48^ While the fitness benefits of circadian rhythms are intuitive, obtaining direct experimental evidence has been challenging. In this study, we established a novel experimental framework to quantify the fitness advantages of *Drosophila* with different free-running periods.

Our experimental design offers two distinct advantages. First, by altering the circadian period within specific brain clock neurons, we minimized pleiotropic effects on other physiological processes, allowing us to attribute the competitive outcomes directly to the circadian activity rhythm itself. Second, the use of eye-color markers enabled the longitudinal tracking of large populations across multiple generations, providing the statistical power necessary to detect subtle fitness differences. We successfully demonstrated that flies with a wild-type (about 24-h) rhythm maintain a clear competitive advantage over those with altered rhythms under LD 12:12 conditions. Remarkably, this advantage was abolished under LL conditions, where the molecular clock is arrested. This result provides compelling evidence that the observed selection pressure is specifically mediated by the circadian system, rather than by pleiotropic effects of *dbt* overexpression or its expression in non-clock cells. Taken together, our findings highlight the critical role of precisely tuned neuronal clocks in optimizing survival and reproductive success.

### Factors contributing to selection pressure

Since our manipulation was restricted to the brain clock neurons while leaving peripheral clocks intact, the observed selection pressure can be primarily attributed to alterations in circadian activity rhythms. Various behaviors, including locomotion, eclosion, mating, feeding, oviposition, and temperature preference, are regulated by the circadian clock.^49^ Given that our flies were maintained under optimal conditions with *ad libitum* food and constant temperature, we hypothesize that the primary competitive stages were eclosion and reproduction.

In theory, flies that eclose earlier may gain a competitive advantage by mating sooner and potentially dominating the subsequent generation. While the adult emergence of *dbt*^*L*^ was slightly delayed—consistent with the fact that circadian period length influences eclosion timing^2^ (Figure 4D)—this factor alone does not fully explain the selection dynamics. Under LD 12:12, both *dbt*^*S*^ and *dbt*^*L*^ declined more rapidly than *dbt*^*WT*^ (Figure 2), indicating that selection against these flies involves more than just eclosion timing.

Reproductive success is a multifaceted process involving mating preference, oviposition, sperm competition, fertilization, and larval development. Following internal competition within the maternal reproductive tract, offspring must navigate further competition in the external environment throughout their developmental stages. Our paternity assays revealed that *dbt*^*WT*^ males possess a clear advantage over both *dbt*^*S*^ and *dbt*^*L*^ (Figure 5), aligning with the results of the long-term competition assays under LD 12:12 (Figure 2). These data suggest that reproductive success is a major factor influencing fitness. It is likely that individuals whose internal clocks are well-synchronized with the LD 12:12 cycle can optimize the timing of their reproductive activities, thereby producing more offspring. Conversely, individuals misaligned with the LD cycle suffer a competitive disadvantage. This behavioral optimization may depend on the connection between different clock tissues. For example, previous studies reported that brain PDF neurons (s-LN_v_s and l-LN_v_s) control the peripheral clocks in oenocytes to regulate mating behavior.^39^ Therefore, the reduced reproductive success in *dbt*^*S*^ and *dbt*^*L*^ flies may be caused not only by mismatched behavioral timing, but also by the miscoupling between brain and peripheral clocks.

Notably, although the hierarchy of competitive advantage in paternity was *dbt*^*WT*^ > *dbt*^*S*^ > *dbt*^*L*^ (Figure 5), *dbt*^*L*^ performed slightly better than *dbt*^*S*^ in the long-term competition assays (Figure 2). This discrepancy suggests that paternity success alone cannot account for the overall survival rates. One contributing factor is likely the experimental design of the paternity assay. By pairing the flies at ZT 12 and ZT 18, we may have forced mating during the night when they typically sleep, which rarely occurs in nature.

Another critical variable is the influence of eye color and the *white* gene on reproductive success. Previous research has shown that *white* (*w*^*1118*^) mutants exhibit reduced mating success compared to wild-type flies, an effect attributed not to eye pigmentation itself but to the pleiotropic roles of the *white* gene in the nervous system.^50^ In our study, the success rate of *dbt*^*WT*^ males (which have red eyes) was highest during the day (ZT 0 and ZT 6) but decreased significantly at night (ZT 12 and ZT 18) (Figure 5). This temporal shift in performance suggests that while eye color or *white* gene function is involved, the overall fitness outcome likely results from a complex interaction between the *white* gene and the circadian system. Importantly, as *dbt*^*WT*^, *dbt*^*S*^, and *dbt*^*L*^ lines share identical eye color, the comparative differences in reproductive success among these three strains can be attributed specifically to their differing circadian rhythms. Therefore, the conclusion that brain circadian rhythms influence competitive fitness remains valid.

### Photoperiod and circadian period

The resonance hypothesis predicts that adaptive value is maximized when an organism’s internal circadian period matches the external environmental cycle.^15^ This concept has been elegantly confirmed in cyanobacteria and *Arabidopsis thaliana*.^23^^,^^25^ In *Drosophila*, however, the resonance hypothesis was only partially supported in *per*^*L*^ mutants and remained unconfirmed in *per*^*S*^ mutants.^21^

In this study, we investigated how circadian periods influence survival under varying photoperiodic conditions. Our results revealed that the relative fitness of *dbt*^*S*^ and *dbt*^*L*^ is dependent on the photoperiod. Under long-day (LD 16:8) conditions, *dbt*^*L*^ maintained a slightly but significantly higher overall frequency than *dbt*^*S*^ (Figure 6). In contrast, under short-day (LD 8:16) conditions, *dbt*^*S*^ exhibited a clear superiority in overall population frequency compared to *dbt*^*L*^ (Figure 7). These results suggest that competitive fitness is determined by the alignment between the internal circadian period and the external photoperiod.

A potential explanation for this difference lies in the temporal distribution and the rhythmicity of their activity. Under LD 8:16, *dbt*^*S*^ activity is primarily restricted to the daytime, with its nocturnal activity suppressed compared to both *w*^*1118*^ and *dbt*^*L*^. Furthermore, the quality of the activity rhythm differed; while *w*^*1118*^ and *dbt*^*L*^ maintained distinct bimodal patterns with clearly defined morning and evening peaks, *dbt*^*S*^ failed to exhibit a sharp morning peak, instead showing more diffuse activity during the second half of night and into the morning. Such well-defined activity peaks are likely crucial for reproductive success, as they synchronize the active windows of males and females, thereby increasing the probability of encounters and successful mating within a population. In contrast, the lack of clear peaks in *dbt*^*S*^, combined with its restricted nocturnal activity, may have limited the effective time for seeking partners. Consequently, while *dbt*^*S*^ may have performed better in the short term due to its higher daytime activity levels, the cumulative disadvantage of a narrower and less synchronized window for reproductive activity likely led to its more rapid decline over multiple generations. In contrast, the delayed evening activity peak of *dbt*^*L*^ might have provided a better temporal fit for nocturnal interactions, mitigating its fitness cost in the long run.

Apart from the early evolutionary history of the Earth, the environmental cycle has consistently remained 24 h. Since light-dark cycles significantly deviating from 24 h are virtually non-existent in nature, it is unlikely that the external period itself has acted as a direct selective pressure. Instead, we argue that the photoperiod exerts a stronger pressure, which aligns closely with recent findings in cyanobacteria demonstrating that the fitness of clock mutants is contingent upon specific photoperiod conditions.^51^ Across latitudinal clines, photoperiod and temperature fluctuate seasonally, requiring insects to adapt to the contrasting environments of summer and winter. Natural populations of *Drosophila melanogaster* exhibit significant variation in free-running periods, including occasional arrhythmicity.^52^ Furthermore, circadian periods often show latitudinal clines,^47^^,^^53^^,^^54^^,^^55^ suggesting that natural selection acts on the intrinsic period as a primary mechanism for phase adjustment to local environments. Laboratory selection experiments for eclosion timing have further demonstrated that the phase of rhythmic behaviors is intimately linked to the internal circadian period.^56^^,^^57^^,^^58^ This natural variety in period length gives the population many options to adapt. Depending on the season or location, the most suitable rhythm is favored, ensuring that the group as a whole stays healthy and successful. This expands the resonance hypothesis: the goal of the internal clock is not just to track a 24-h cycle, but to ensure that activity peaks are perfectly timed for the specific day length.

### Limitations of the study

Although our results show that the brain’s pacemaker clock confers a clear fitness advantage, several caveats should be noted. First, while the *DvPdf* promoter is relatively specific to pacemaker neuron subsets, its expression is not entirely restricted to clock cells. Since *dbt* is involved in various developmental processes beyond just circadian regulation,^59^^,^^60^^,^^61^^,^^62^ its overexpression might exert subtle, clock-independent effects on survival that were difficult to isolate. Confirming these findings across different genetic backgrounds remains an important next step.

Second, as demonstrated by our paternity assays, eye color also influences reproductive success. Because the *dbt* transgenic lines (marked with *mini-white*) competed directly against *w*^*1118*^ mutants, the survival outcomes likely reflect a combination of circadian timing and eye color. While this factor complicates the strict isolation of clock-specific traits, addressing this with a more neutral genetic marking method will be a key challenge for future studies.

## Resource availability

### Lead contact

Further information and requests for resources and reagents should be directed to and will be fulfilled by the lead contact, Taishi Yoshii (yoshii@okayama-u.ac.jp).

### Materials availability

All fly strains generated in this study are available from the lead contact upon request.

### Data and code availability

• Data reported in this study will be shared by the lead contact upon request. Accession numbers are listed in the key resources table.
• This study does not report original code.
• Any additional information required to reanalyze the data reported in this article is available from the lead contact upon request.

## Acknowledgments

We thank Jeffrey Price and the Developmental Studies Hybridoma Bank for providing materials and reagents, Nils Reinhard for valuable comments on the manuscript, and Yuuki Konishi for technical assistance with the lifespan experiments. Fly stocks obtained from the Bloomington Drosophila Stock Center (NIH P40OD018537) were used in this study. This work was supported by 10.13039/501100001691JSPS 10.13039/501100001691KAKENHI grant number 24K09534. This work has been partly supported by Core-Facility at Okayama University (CFPOU DIA_717).

## Author contributions

Conceptualization, T.Y.; methodology, T.Y. and S.A.; formal analysis, M.M. and S.A.; investigation, S.A. and S.T.; resources, T.Y. and S.T.; writing – original draft, T.Y.; writing – review and editing, M.M. and S.A.; visualization, S.A. and T.Y.; funding acquisition, T.Y.

## Declaration of interests

The authors declare no competing interests.

## Declaration of generative AI and AI-assisted technologies in the writing process

During the preparation of this work the authors used Gemini in order to improve the readability and language of the manuscript. After using this tool/service, the authors reviewed and edited the content as needed and take full responsibility for the content of the published article.

## STAR★Methods

### Key resources table

**Table:** 

REAGENT or RESOURCE	SOURCE	IDENTIFIER
**Antibodies**		

Chicken anti-GFP	Rockland	Cat# 600-901-215; RRID: AB_1537402
Mouse anti-PDF	Developmental Studies Hybridoma Bank	RRID: AB_760350
Alexa Fluor 488 (goat anti-chicken IgG (=IgY))	Thermo Fisher Scientific	Cat# A11039; RRID: AB_2534096
Alexa Fluor 555 (donkey anti-mouse IgG)	Thermo Fisher Scientific	Cat# A31570; RRID: AB_2536180

**Chemicals, peptides, and recombinant proteins**		

Vectashield mounting medium	Vector Laboratories	Cat# H-1000
PrimeSTAR Max DNA Polymerase	Takara Bio Inc.	Cat# R045A
In-Fusion HD enzyme	Takara Bio Inc.	Cat# 639648
Normal donkey serum	Sigma-Aldrich	Cat# D9663-10 ML

**Experimental models: Organisms/strains**		

*D*. *melanogaster*: *w*^*1118*^ control strain	Bloomington Drosophila Stock Center	BDSC: 5905; RRID: BDSC_5905
*D*. *melanogaster*: *13xLexAop2-mCD8*:*:GFP*	Bloomington Drosophila Stock Center	BDSC: 32203; RRID: BDSC_32203
*D*. *melanogaster*: *w*^*1118*^*;;DvPdf-dbt*^*WT*^ (*dbt*^*WT*^)	This paper	N/A
*D*. *melanogaster*: *w*^*1118*^*;;DvPdf-dbtS* (*dbt*^*S*^)	This paper	N/A
*D*. *melanogaster*: *w*^*1118*^*;;DvPdf-dbtL* (*dbt*^*L*^)	This paper	N/A
*D*. *melanogaster*: *w*^*1118*^*;;DvPdf-LexA*	This paper	N/A

**Oligonucleotides**		

Forward primer for *DvPdf-LexA*: TTCGTCTTCAAGAATGTACCAGGACGATTCTTGA	This paper	N/A
Reverse primer for *DvPdf-LexA*: TTTATCACCACTTTAACTTGGGAATGAACAAATGC	This paper	N/A
Forward primer for *DvPdf-dbt*: CCTTTCGTCTTCAAGGTACCAGGACGATTCTTGA	This paper	N/A
Reverse primer for *DvPdf-dbt*: CGCAGCTCCATTTTGAACTTGGGAATGAACAAATG	This paper	N/A
Forward primer for *dbt* amplification: CAAAATGGAGCTGCGCGT	This paper	N/A
Reverse primer for *dbt* amplification: CCGGTACCAGATCTGTTATTTGGCGTTCCCCACG	This paper	N/A
Forward primer for *pBPLexA*;;*p65Uw* inverse PCR: AAAGTGGTGATAAACGGCCG	This paper	N/A
Reverse primer for *pBPLexA*;;*p65Uw* inverse PCR: ATTCTTGAAGACGAAAGGGC	This paper	N/A
Forward primer for *pBDP inverse* PCR: CAGATCTGGTACCGGATCCCC	This paper	N/A
Reverse primer for *pBDP* inverse PCR: CTTGAAGACGAAAGGGCCTCG	This paper	N/A

**Recombinant DNA**		

Plasmid: pBPLexA;p65Uw	Addgene	Addgene Plasmid #26231
Plasmid: pBDP	Addgene	Addgene Plasmid #17566
Plasmid: *DvPdf-gal4*	Prof. Jae H. Park	N/A
Plasmid: *UAS-dbt*^*WT*^	Prof. Jeffrey L. Price	N/A
Plasmid: *UAS-dbt*^*S*^	Prof. Jeffrey L. Price	N/A
Plasmid: *UAS-dbt*^*L*^	Prof. Jeffrey L. Price	N/A
Plasmid: *DvPdf-LexA*	This paper	N/A
Plasmid: *DvPdf-dbt*^*WT*^	This paper	N/A
Plasmid: *DvPdf-dbt*^*S*^	This paper	N/A
Plasmid: *DvPdf-dbt*^*L*^	This paper	N/A

**Software and algorithms**		

ImageJ	Schneider et al.^63^	https://imagej.net/ij/
ActogramJ	Schmid et al.^64^	https://bene51.github.io/ActogramJ/
R version 4.5.1	The R Project	https://www.r-project.org/

**Other**		

Drosophila Activity Monitor (DAM2)	Trikinetics Inc.	N/A
Incubator (CN-40 A)	Mitsubishi Electric	N/A
Incubator (MIR-153)	PHCbi	N/A
Confocal Microscope (FV3000)	Olympus	N/A

### Experimental model and study participant details

#### Fly strains

Flies were reared at 25°C under a light dark cycle with LD12:12 on *Drosophila* medium (0.7% agar, 8.0% glucose, 3.3% yeast, 4.0% cornmeal, 2.5% wheat embryo, and 0.25% propionic acid). *white*^*1118*^ (*w*^*1118*^; Bloomington *Drosophila* Stock Center [BDSC] #5905) strain was used as a control strain in this study. Additionally, the following strains were used: *13xLexAop2-mCD8*:*:GFP* (BDSC #32203), *w*^*1118*^*;;DvPdf-dbt*^*WT*^ (*dbt*^*WT*^), *w*^*1118*^*;;DvPdf-dbt*^*S*^ (*dbt*^*S*^), *w*^*1118*^*;;DvPdf-dbt*^*L*^ (*dbt*^*L*^), and *w*^*1118*^*;;DvPdf-LexA*. To minimize the effects of genetic background, the *dbt*^*WT*^, *dbt*^*S*^ and *dbt*^*L*^ strains were outcrossed at least six times into *w*^*1118*^ control flies.

### Method details

#### Generation of *DvPdf* transgenic lines

The *DvPdf* promoter sequence from the *DvPdf-gal4* plasmid vector (kindly provided by Prof. Jae H. Park) was amplified by Polymerase Chain Reaction (PCR) using Forward primer: TTCGTCTTCAAGAATGTACCAGGACGATTCTTGA and Reverse primer: TTTATCACCACTTTAACTTGGGAATGAACAAATGC for *DvPdf-LexA*, and Forward primer: CCTTTCGTCTTCAAGGTACCAGGACGATTCTTGA and Reverse primer: CGCAGCTCCATTTTGAACTTGGGAATGAACAAATG for *DvPdf-dbt*. The *dbt*^*WT*^, *dbt*^*S*^, and *dbt*^*L*^ sequences were amplified from *UAS-dbt*^*WT*^, *UAS-dbt*^*S*^, and *UAS-dbt*^*L*^ vectors (kindly provided by Prof. Jeffrey L. Price) using Forward primer: CAAAATGGAGCTGCGCGT and Reverse primer: CCGGTACCAGATCTGTTATTTGGCGTTCCCCACG. These DNA fragments were cloned into *pBPLexA;;p65Uw* (addgene #26231) or *pBDP* (addgene #17566). *pBPLexA;;p65Uw* and *pBDP* vectors were linearized by inverse PCR using Forward primer: AAAGTGGTGATAAACGGCCG and Reverse primer: ATTCTTGAAGACGAAAGGGC for *pBPLexA;;p65Uw*, and Forward primer: CAGATCTGGTACCGGATCCCC and Reverse primer: CTTGAAGACGAAAGGGCCTCG for *pBDP*. Prime STAR Max DNA Polymerase (Takara Bio Inc.) was used for the above PCR. The DNA fragments were ligated with In-Fusion HD enzyme (Takara Bio Inc., Japan) and subsequently transformed into *E*. *coli* competent cells. The Plasmid DNA of *dbt*^*WT*^, *dbt*^*S*^, *dbt*^*L*^, and *DvPdf-LexA* were purified and injected into *Drosophila* embryos (BestGene Inc.). All constructs were inserted into the same genomic region (3 R, ZH-86Fb) using PhiC31 integrase-mediated transgenesis systems.

#### Activity recording and data analysis

Male flies aged 3–4 days were used to record locomotor activity rhythms. Flies were confined to recording tubes containing agar/sugar food (2% agar and 4% sucrose) for the *Drosophila* Activity Monitor (DAM2; Trikinetics Inc., Waltham, MA, USA). The monitors were placed in an incubator (CN-40 A, Mitsubishi Electric, Tokyo, Japan) maintained at a constant temperature of 25°C. Standard cool white LEDs were set above the monitors in the incubator and controlled by an LC4 light controller (Trikinetics Inc.). The light intensity used in all experiments was 100 lux (32 μW cm^−2^ for the white LEDs). For visual inspection, raw data were visualized as actograms using ActogramJ.^64^ The activity of flies was recorded under LD12:12 for eight days, followed by DD or LL. Average daily activity patterns were calculated using data from Days 4–8 (5 days) for the LD 12:12 condition and Days 10–15 (6 days) for the LL condition. To assess the LD rhythm after the flies were fully re-entrained to the new conditions, the first three days were excluded from the calculations. To determine the phases of activity peaks, PHASE analysis was performed using data from Days 4–8.^65^^,^^66^ To determine free-running periods and rhythm strength (power), we performed Chi-square periodogram analysis^67^ on locomotor activity data from Days 9–18 (9 days) in DD. A significance threshold of *p* < 0.01 was applied to define rhythmicity.

#### Immunohistochemistry

Whole flies were fixed in 4% paraformaldehyde in phosphate-buffered saline (PBS) with 0.1% Triton X-100 at room temperature (RT, around 25°C) for 2.5 h. The fixed flies were washed three times with PBS, and their brains and ventral nerve cords were dissected. They were then washed three times with PBS containing 0.5% Triton X-100 (PBS-T), blocked in PBS-T containing 5% normal donkey serum for 1 h at RT and subsequently incubated with primary antibodies at 4°C for 48 h. After washing six times with PBS-T, the brains were incubated with secondary antibodies at RT for 3 h, washed six more times in PBS-T, and mounted in Vectashield mounting medium (Vector Laboratories, Burlingame, CA, USA). The primary antibodies used were as follows: chicken anti-GFP (1:1000; Rockland, Limerick, PA, United States), and mouse anti-PDF (1:500; Developmental Studies Hybridoma Bank).^68^ We used the following fluorescence-conjugated secondary antibodies at a 1:1000 concentration: Alexa Fluor-488 (goat anti-chicken), and Alexa Fluor-555 nm (donkey anti-mouse). Staining was visualized using a laser scanning confocal microscope (FV3000; Olympus, Tokyo, Japan).

#### Competition assay

*w*^*1118*^ control flies were co-housed with *dbt*^*WT*^, *dbt*^*S*^, or *dbt*^*L*^ transgenic lines in the same vials for 27 generations (Figure S3). All vials were maintained in an incubator (MIR-153, PHCbi, Tokyo, Japan) to control a constant temperature of 25°C and LD12:12 or constant light (LL) conditions (approximately 100–200 lux for cool white LEDs). Before initiating the competition assay, *w*^*1118*^ virgin females were mated with males of each *dbt* strain for 3 days. Subsequently, the mated *w*^*1118*^ females were transferred to new vials. After seven days, the females that had laid eggs were removed from the vials, and *w*^*1118*^*;;dbt/* + heterozygous flies were obtained. The competition assay was initiated with these *dbt/* + heterozygous flies. This approach was chosen to minimize artificial founder effects and reduce stochastic genetic drift in the first generation, thereby ensuring that subsequent allele frequency changes were predominantly driven by selection rather than by initial sampling bias. Newly emerged adult flies were transferred into new vials, where they freely mated and laid eggs for seven days. Subsequently, the adult flies were removed, and after seven days, newly emerged adult flies were transferred into new vials. This process was repeated for 27 generations. The number of each genotype of male flies was counted every three generations. Since the transgenic flies possess a *mini-white* marker gene, *dbt* homozygous strains exhibit red eyes, *dbt* heterozygous strains orange eyes, and strains lacking *dbt* white eyes. For counting, only male flies were used to minimize effects of anesthesia on females. After 27 generations, a subset of flies from each genotype was randomly selected, and their activity rhythms were recorded to confirm the correspondence between eye colors and free-running rhythms.

#### Lifespan

Lifespan was measured with five vials for each genotype, in which 30 adult male flies were housed in one vial. The flies were maintained in an incubator to control a constant temperature of 25°C and LD12:12 conditions (approximately 100–200 lux for cool white LEDs). The number of surviving flies was counted every two days, at which time flies were transferred into new vials.

#### Number of offspring and development period

Four virgin adult female and four adult male flies of the same genotypes were housed together in the same vials in an incubator under a constant temperature of 25°C and LD12:12 conditions (approximately 100 lux for cool white LEDs) at ZT6. After 48 h, all adult flies were removed from the vials. From day 10 to day 14, the number of newly emerged adult offspring of each genotype was counted daily.

#### Paternity assay

Virgin adult female flies and adult male flies were entrained in LD 12:12 or LL conditions prior to the experiments. At each zeitgeber time (ZT), four females (either *w*^*1118*^ or *dbt*), two *w*^*1118*^ males, and two *dbt* homozygous males were housed together in the same vials and maintained in an incubator under a constant temperature of 25°C and LD12:12 conditions (approximately 100 lux for cool white LEDs). For assays involving *dbt* homozygous females, the genotype of the *dbt* homozygous males was matched to that of the females (e.g., *dbt*^*WT*^ homozygous males were used for *dbt*^*WT*^ homozygous females). Following a 48-h mating and oviposition period, the parental flies were removed, and the relative reproductive success of each male genotype was estimated based on the proportion of adult offspring produced. From day 10 to day 14, the number of newly emerged adult offspring of each genotype was counted daily. Paternity was determined by the genotype of the offspring. In crosses with *w*^*1118*^ females, offspring were identified as white-eyed (*w*^*1118*^-sired) or orange-eyed (*dbt*-sired). In crosses with *dbt* females, offspring were identified as orange-eyed (*w*^*1118*^-sired) or red-eyed (*dbt*-sired).

### Quantification and statistical analysis

To compare the period and power of activity rhythms, and the phases of the M and E peaks obtained from PHASE analysis across fly strains, we performed the Shapiro-Wilk test to assess normality. Based on the results, we used either one-way ANOVA with Tukey’s post-hoc test, or the Kruskal-Wallis test followed by pairwise Mann-Whitney U tests with Holm’s correction. All of the statistical details of experiments can be found in the figures and figure legends. Statistical significance was defined as *p* < 0.01. Significant differences are indicated by asterisks in Figure S2A (∗∗*p* < 0.01) and by different letters in Figures 1E, 6B, and 7B. These statistical analyses were conducted using R version 4.5.1.

Changes in transgenic allele (*dbt*^*X*^) frequencies in long-term competition assays against the control strain were modeled using generalized linear mixed models (GLMMs) assuming a binomial distribution (logit link), including strain, generation, and their interaction as fixed effects, and experimental replicates as random intercepts. The generation was mean-centered so that the intercept corresponded to the average sampled generation. This modeling framework allowed us to estimate both the average differences in allele frequency and their change over time, while accounting for the non-independence of repeated measurements from the same lineages and for stochastic, replicate-specific fluctuations that would otherwise bias inference on allelic dynamics during competition. The estimated marginal means for each strain, averaged over the other fixed effects in the model, were obtained from the GLMM using the emmeans package in R version 4.5.1. Pairwise contrasts among strains were assessed using z-tests on the GLMM-based estimated marginal means, with results reported as odds ratios. The resulting *p*-values were adjusted for multiple comparisons using Holm’s method. In addition, deviations of each strain’s estimated marginal mean from the expected 0.5, which is an initial allele frequency at generation 1, were evaluated using one-sample z-tests on the logit scale against the null of logit(0.5). All of the statistical details of experiments can be found in the figures and figure legends. Significant differences in allele frequencies among genotypes are indicated by different letters in Figures 2C, 3C, 6D, and 7D (*p* < 0.05).

Paternity was quantified as the number of offspring sired by transgenic males and analyzed using generalized linear mixed models (GLMMs) with a binomial error distribution (logit link). The models included strain, ZT at the start of mating, and their interaction as fixed effects, and the experimental replicate as a random intercept. Within each ZT, pairwise contrasts among strains were assessed using z statistics from estimated marginal means obtained on the logit scale and back-transformed to the response scale, with *p*-values adjusted for multiple comparisons using Holm’s correction. All of the statistical details of experiments can be found in the figures and figure legends. Significant differences in offspring paternity frequencies among genotypes are indicated by different letters in Figures 5 and S5 (*p* < 0.05).
