# Supplementary material for: Brain circadian clock neurons drive fitness advantages in Drosophila
Source: iScience. 2026 Aug 10;29(8):117125. doi: 10.1016/j.isci.2026.117125 (PMC13486361; doi:10.1016/j.isci.2026.117125)
Supplement: Supplementary file 1 [file mmc1.pdf]

iScience, Volume *29*

Supplemental information

Brain circadian clock neurons drive fitness advantages in *Drosophila*

Sae Aikawa, Shoichiro Tamura, Makiko Mimura, and Taishi Yoshii

Figure S1

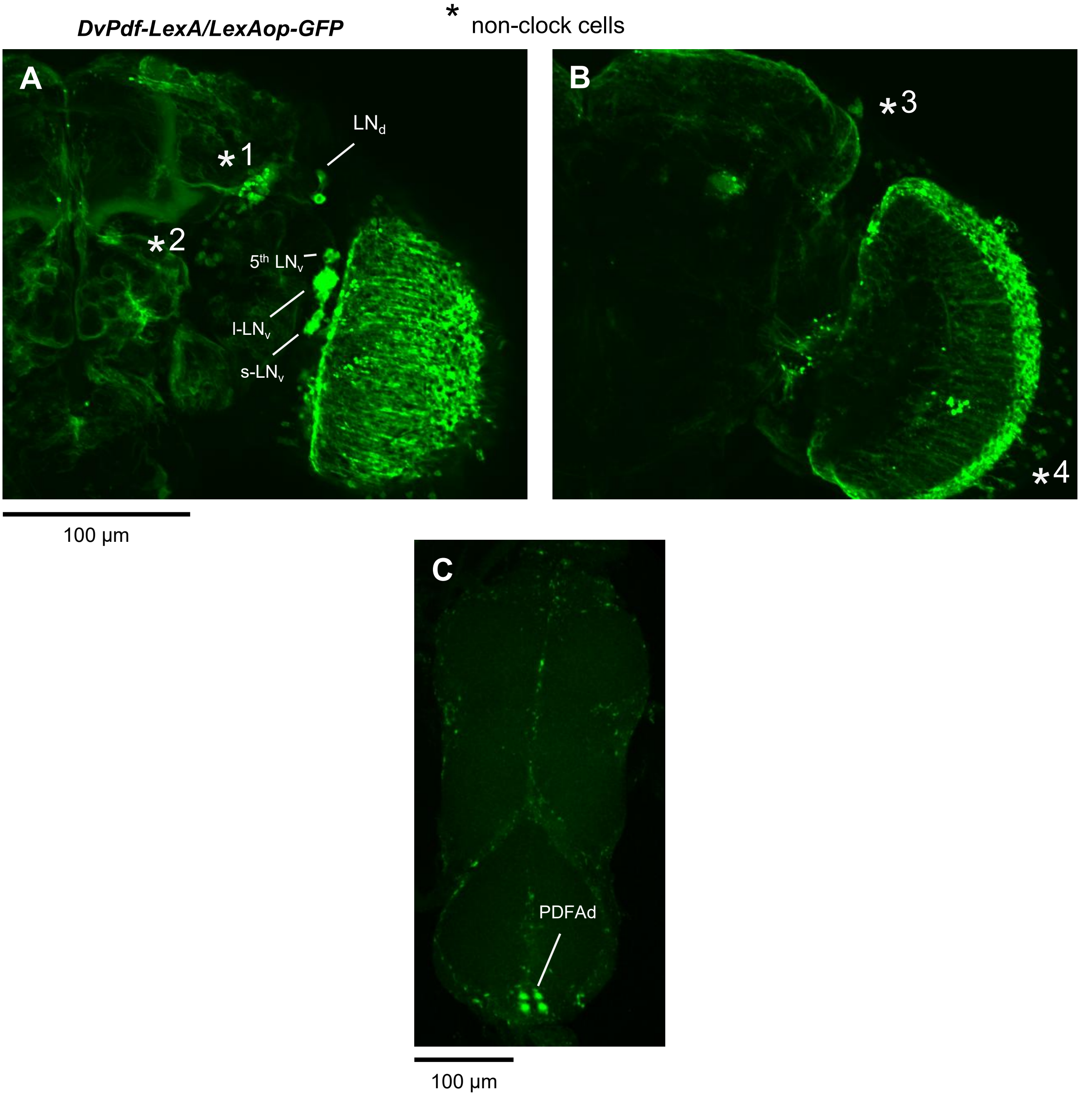

Figure S1. Expression patterns of the *DvPdf-LexA/LexAop-GFP* line in the adult brain and ventral nerve cord.
(A) GFP expression is localized to the s-LN$_v$s, l-LN$_v$s, 5th LN$_v$, and LN$_d$s, as well as several non-clock cell clusters (indicated by asterisks). One non-clock cluster exhibiting a robust GFP signal is situated near the mushroom body (asterisk 1), while another is located lateral and ventral to the mushroom body (asterisk 2). **(B)** This image represents a projection of Z-stacks taken at a different focal depth than that in (A). A third non-clock cluster (asterisk 3) is visible, showing weak GFP expression. Another weak cluster (asterisk 4) is located along the rim of the optic lobes. **(C)** Four PDF-expressing abdominal neuromeres (PDFAb) neurons are identified in the ventral nerve cord. Scale bar = 100 µm.

Figure S2

A

Strain	Period ± SEM	Power ± SEM	R (%)	Complex (%)	n
w^{1118}	24.06 ± 0.06	176.27 ± 9.67	100	0	29
dbt^{WT}	24.43 ± 0.04	163.07 ± 8.50	100	0	23
dbt^{S}	17.68 ± 0.08**	100.17 ± 5.8**	81.48	8	27
dbt^{L}	26.94 ± 0.08**	174.08 ± 8.18	100	0	27

B

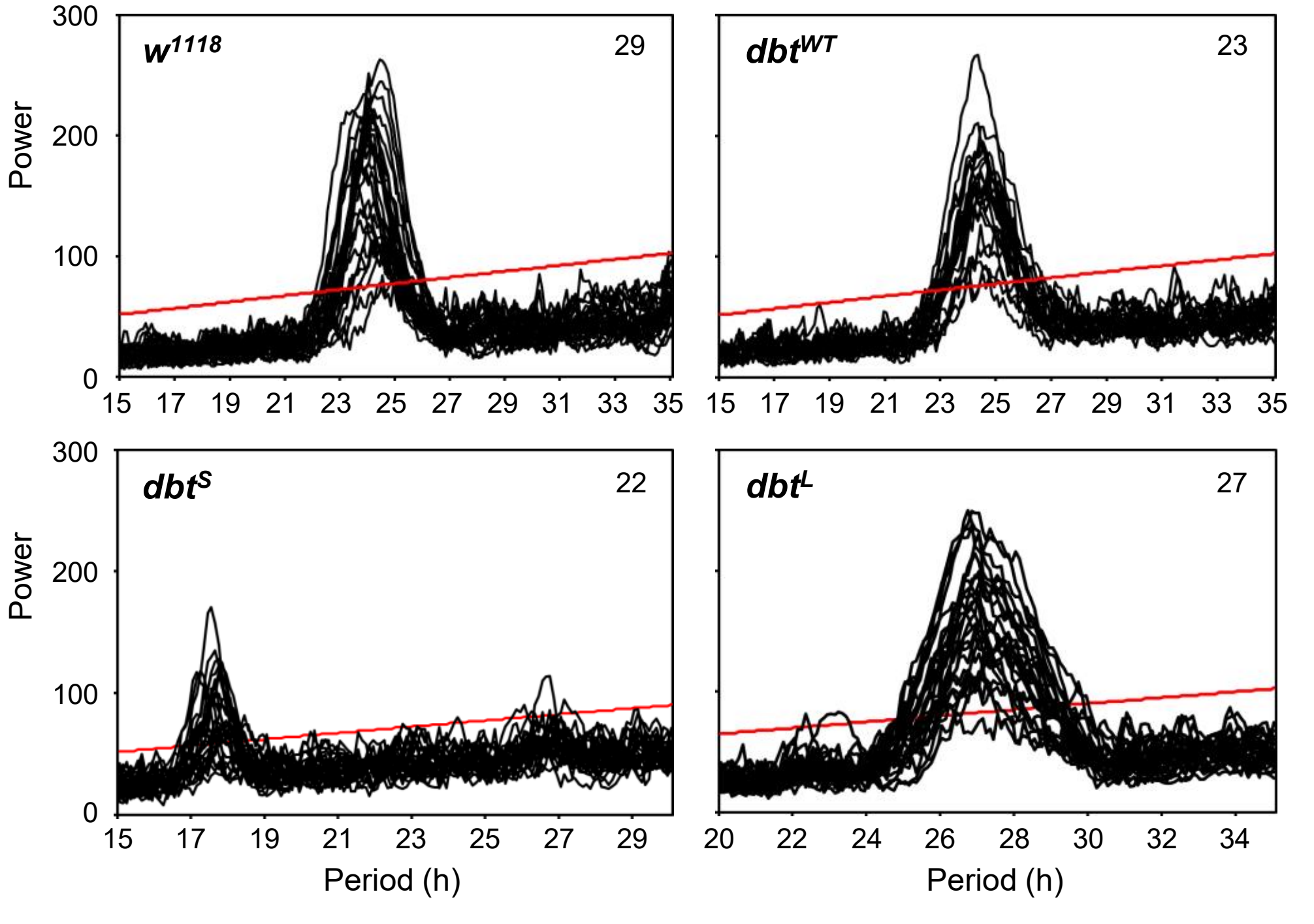

Figure S2. Free-running rhythms of *dbt* lines under DD conditions.
(A) Free-running periods in DD (presented in Fig. 1C) were determined using Chi-square periodogram analysis. Periods of both dbt^{S} and dbt^{L} flies were significantly different from dbt^{WT} controls ($p < 0.01$, Kruskal-Wallis test with Holm's correction for period; one-way ANOVA with Tukey's multiple comparison test for power). The percentage of rhythmic individuals was lower in dbt^{S} flies than in other lines, with a small number of dbt^{S} flies exhibiting complex rhythms. See also Fig. 1C. **(B)** Chi-square periodogram profiles for all tested individuals. Although the power of dbt^{S} flies was lower compared to that of w^{1118}, dbt^{WT}, and dbt^{L} flies, most dbt^{S} individuals exhibited consistently shorter free-running periods. Individual black lines indicate the results of the Chi-square periodogram analysis, and red lines indicate the significance threshold ($p < 0.01$).

Figure S3

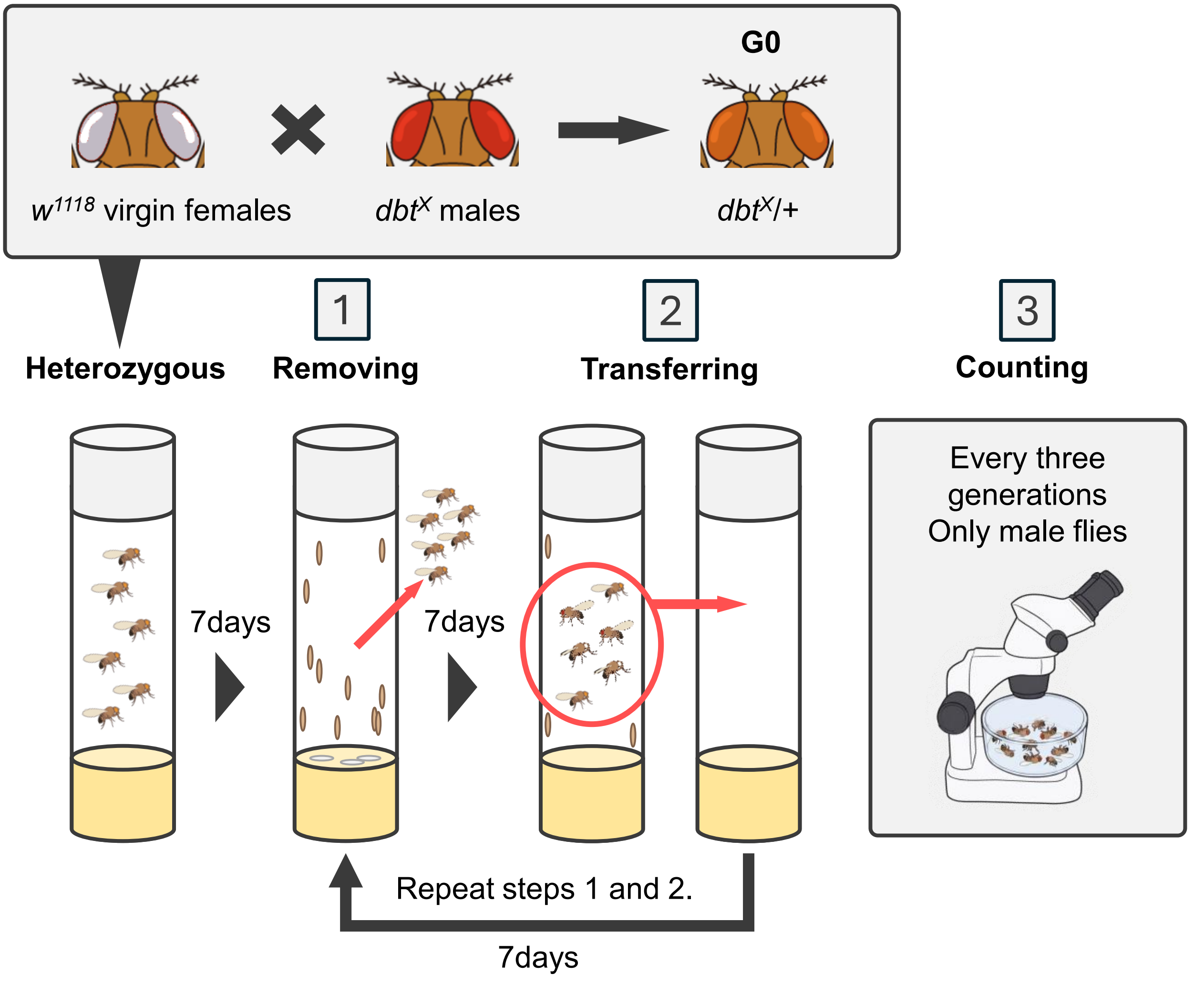

Figure S3. Experimental design for long-term competition assays.
Virgin w^{1118} females were first mated with males of each *dbt* strain to obtain heterozygous offspring. The competition assay was initiated using these *dbt* heterozygotes. Newly emerged adult flies were transferred into new vials, where they were allowed to mate freely and lay eggs for seven days. Subsequently, the parental adults were removed **(1)**. After another seven days, the newly emerged next-generation adults were transferred to new vials **(2)**; this cycle was repeated for 27 generations. The number of male flies for each genotype was counted every three generations **(3)**. Because the transgenic lines carry a *mini-white* marker, eye color phenotypes distinguish the genotypes: *dbt* homozygotes exhibit red eyes, heterozygotes exhibit orange eyes, and individuals lacking the *dbt* transgene exhibit white eyes.

Figure S4

A

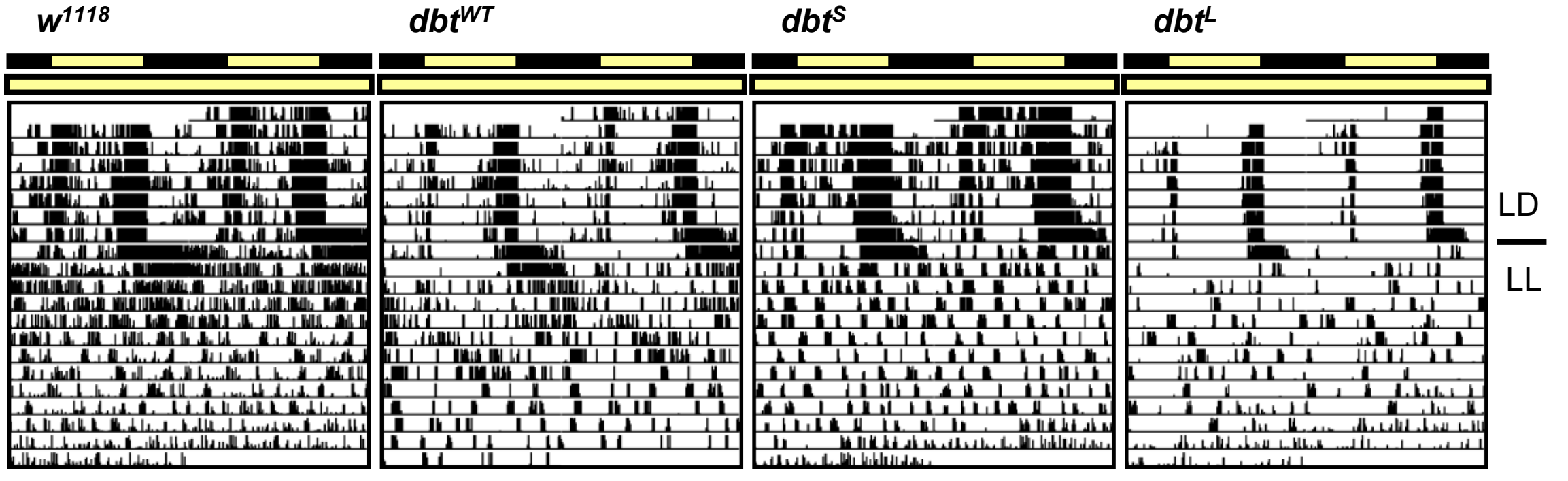

B

Strain	Period ± SEM	Power ± SEM	R (%)	Complex (%)	n
w^{1118}			0	0	28
dbt^{WT}	30.30 ± 0.99	115.45 ± 3.13	9.09	0	22
dbt^{S}	28.15 ± 2.58	94.59 ± 11.47	8	0	25
dbt^{L}	22.4	117.33	9.09	9.09	22

Figure S4. Locomotor activity of *dbt* flies under LL conditions
(A) Locomotor activity rhythms under LD 12:12 and constant light (LL). Representative actograms are shown for *w*^{1118} and *dbt* transgenic lines. Flies were entrained for seven days under LD 12:12 before being transferred to LL. **(B)** Chi-square periodogram analysis for free-running periods in LL. Most individuals across all tested lines exhibited arrhythmicity under LL conditions.

Figure S5

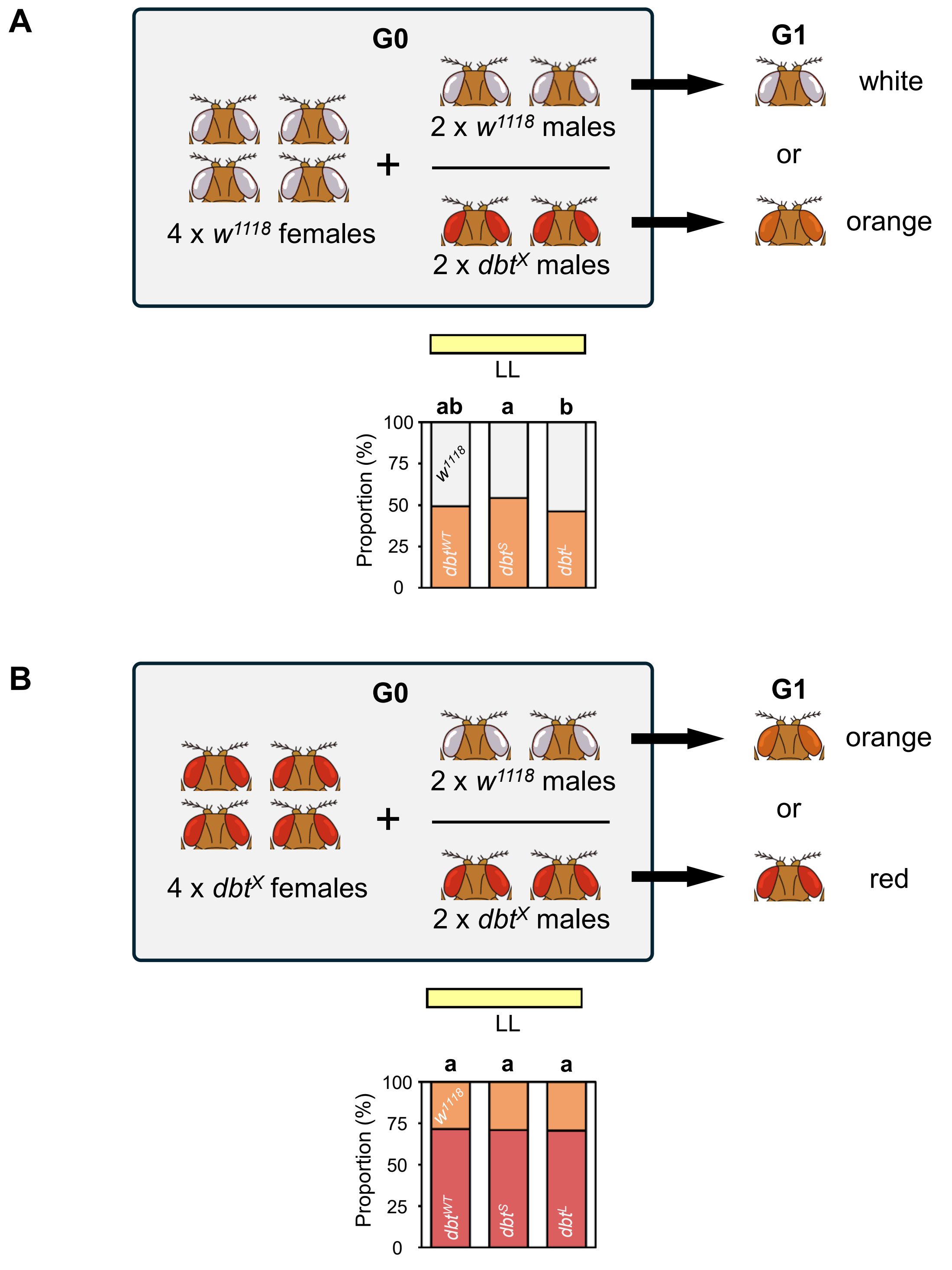

Figure S5. Male reproductive success (paternity) under LL conditions
(A) Proportion of offspring sired by w^{1118} or *dbt* homozygous males when paired with w^{1118} females under constant light (LL) conditions. G0 parental flies were reared under LL throughout development prior to mating. Assays were initiated by pairing four females with two w^{1118} and two *dbt* homozygous males at 12:00 PM (n = 11 vials per genotype). **(B)** Proportion of offspring sired when paired with *dbt* homozygous females under LL conditions (n = 12 vials per genotype). For both panels, different letters indicate statistically significant differences in genotype frequencies ($p < 0.05$, GLMM followed by pairwise comparisons with Holm's adjustment).
